# Titanium-Integrated Magnetic Silica Aerogels via Microfluidic Synthesis for Pesticide Removal from Water

**DOI:** 10.3390/gels12040309

**Published:** 2026-04-03

**Authors:** Elena-Theodora Moldoveanu, Adelina-Gabriela Niculescu, Dana-Ionela Tudorache (Trifa), Alexandra-Cătălina Bîrcă, Bogdan Purcăreanu, Ionela C. Voinea, Miruna S. Stan, Bogdan-Ștefan Vasile, Dan Eduard Mihaiescu, Tony Hadibarata, Alexandru Mihai Grumezescu

**Affiliations:** 1 Department of Science and Engineering of Oxide Materials and Nanomaterials, National University of Science and Technology POLITEHNICA Bucharest, 1-7 Polizu Street, 011061 Bucharest, Romania; elena.moldoveanu99@upb.ro (E.-T.M.); adelina.niculescu@upb.ro (A.-G.N.); alexandra.birca@upb.ro (A.-C.B.); bogdanpb89@gmail.com (B.P.); tony.hadibarata@upb.ro (T.H.); 2 Research Institute of the University of Bucharest—ICUB, University of Bucharest, 90-92 Panduri, 050663 Bucharest, Romania; 3 BIOTEHNOS SA, Gorunului Rue, No. 3-5, 075100 Otopeni, Romania; 4 Department of Biochemistry and Molecular Biology, Faculty of Biology, University of Bucharest, 91-95 Splaiul Independentei, 050095 Bucharest, Romania; ionela-cristina.voinea@bio.unibuc.ro (I.C.V.); miruna.stan@bio.unibuc.ro (M.S.S.); 5 Research Center for Advanced Materials, Products and Processes, National University of Science and Technology POLITEHNICA Bucharest, 060042 Bucharest, Romania; bogdan.vasile@upb.ro; 6 National Research Center for Micro and Nanomaterials, National University of Science and Technology POLITEHNICA Bucharest, 060042 Bucharest, Romania; 7 Department of Organic Chemistry, National University of Science and Technology POLITEHNICA Bucharest, 011061 Bucharest, Romania; danedmih@gmail.com; 8 Environmental Engineering Program, Faculty of Engineering and Science, Curtin University Malaysia, CDT 250, Miri 98009, Malaysia

**Keywords:** magnetic aerogel, silica aerogel, iron oxide nanoparticles, titanium, microfluidic synthesis, water decontamination, pesticides adsorption, environmental remediation

## Abstract

Pesticides are a major cause of water contamination, making this issue a major environmental and public health concern. In this context, the development of advanced and effective remediation materials is needed. In this study, a titanium-functionalized magnetic silica aerogel (AG-Ti@Fe_3_O_4_-SA) was successfully prepared via microfluidics and evaluated for water decontamination. The structural and compositional features of the aerogel were determined using XRD, FT-IR, RAMAN, SEM, TEM, BET, and DLS, confirming the formation of the aerogel with dispersed Fe_3_O_4_-SA nanoparticles and the successful incorporation of titanium within the aerogel matrix. Regarding decontamination potential, the aerogel was tested against a pesticide mixture, yielding pesticide-dependent removal efficiencies (16–100%). Notably, the aerogel exhibited a high affinity for organophosphorus pesticides and a moderate affinity for polar compounds, whereas bulky hydrophobic pesticides showed lower adsorption. In vitro, the aerogel induced a moderate decrease in HaCaT cell viability after 48 h of exposure, accompanied by a slight increase in lactate dehydrogenase release, while HEK293 cells remained largely unaffected, indicating a cell-type-dependent biological response. Overall, the findings from this screening-level study recommend AG-Ti@Fe_3_O_4_-SA aerogel as a promising selective adsorbent for pesticide removal.

## 1. Introduction

Water is an essential element for life, used in food production and involved in economic development. However, water pollution poses a global burden, associated with widespread diseases and mortality. In this context, the need for safe and accessible water is urgent [1,2,3,4,5]. Water pollution mainly results from industrialization, which releases organic and inorganic substances, solvents, and volatile chemicals into water bodies [1,6,7,8]. Another area that impacts water pollution is agriculture, as pesticides, nitrogen fertilizers, and organic farm waste significantly degrade water quality [2,7,8]. Conventional water treatment methods are limited, often requiring high energy use or incomplete pollutant removal, underscoring the need for new, effective solutions [6,9]. In this regard, researchers started to investigate nanotechnology approaches that lead to advanced adsorptive materials such as polymer nanomaterials, carbon nanotubes, (nano) zeolites, carbon, graphene, metallic NPs, graphene quantum dots, and aerogels [5,6,10].

Aerogels represent a class of three-dimensional nanotructured materials characterized by high micro- and nanoporosity and very large specific surface area [11,12]. Because of their specific characteristics and properties, fabricating composite aerogels enables new features that allow property tailoring, leading to improvements in water affinity, mechanical strength, and specific applications with enhanced performance [11]. Aerogels have remarkable properties, such as micro- and nanoporous structures, making them suitable as sorbents for water treatment. Thus, the literature reports different types of aerogels for efficient removal of contaminants, including metals, pharmaceutical compounds, oil, fertilizers, dyes, and organic solvents [13,14]. Regarding water decontamination, the literature reports a wide range of aerogels based on alginate, biochar, cellulose, chitosan, gelatin, carbon, metal–organic frameworks, polymers, and silica [12]. Moreover, the most used material in aerogel fabrication is silica, due to its properties that can be improved by organic–inorganic hybridization [13,15].

Silica-based aerogels have immense potential for applications addressing the pressing global challenge of water pollution, serving as suitable sorbents for various contaminants, including dyes, heavy metals, pesticides and herbicides, phenolic compounds, and pharmaceuticals [16,17,18]. One concerning pollution issue is pesticide pollution. Due to their diversified physical and chemical nature, their removal from water bodies is hindered [19]. In this context, some of the literature reports several studies that address this issue. For example, El-Said et al. [20] developed a silica-based composite for the removal of chloridazon (a pyridazinone herbicide) from aqueous media. The composite demonstrated strong performance in removing chloridazon from water (90%) through physical interactions. Also, composite materials based on carbon and iron oxide can be efficiently used to address the pesticide issue [21]. El-Sheikh et al. [22] evaluated the adsorptive capacity of multi-walled carbon nanotubes, C18 silica, and activated carbon as separate sorbents towards pesticides. Silica C18 demonstrated good analytical performance for pesticide extraction, but its efficiency was considerably lower than that of carbon-based materials for all three pesticides investigated (atrazine, propoxur, and methidathion). Roostaie et al. [23] developed an aerogel based on nanoporous, modified silica for the extraction of chlorobenzenes from water samples. Moreover, Feng et al. [24] evaluated the possibility of magnetic silica aerogel for the adsorption of pyrethroid insecticides. However, beyond our previous studies, the literature provides limited information on the extraction of the pesticides exemplified and used in this study.

Introducing magnetic adsorbents, such as magnetite (Fe_3_O_4_)-based materials, can provide distinct advantages, such as strong adsorption activity, easy recovery from aqueous media using an external magnetic field, and efficient magnetic separation [25,26]. In this context, magnetite’s adsorptive properties are attributed to its highly active surfaces, which can bind both positively and negatively charged ions [27]. When incorporated into aerogel composites, their high magnetism, adsorption performance, biodegradability, and reusability make them particularly attractive and cost-effective for water purification applications [28].

Moreover, material features can be improved through the synthesis route. Microfluidics is a versatile approach to synthesizing nanomaterials, enabling high-precision synthesis by controlling reaction parameters to optimize morphology and size, thereby improving monodispersity, production efficiency, and quality [29,30,31]. Microfluidic synthesis promotes a precise manipulation of fluids in confined microchannels. Thus, microfluidics can be a versatile approach for obtaining nanomaterials due to controlled hydrodynamic mixing and continuous reaction conditions. The microscale reaction environment can improve mass transfer compared to conventional reaction environments, leading to controlled precursor mixing, reaction equilibrium, and nucleation growth process. Moreover, during microfluidic synthesis, parameters such as flow rates, channel geometry, and residence time help control reaction conditions and material formation. Additionally, this synthesis strategy enables sequential reactions to assemble complex nanostructures. As a result, microfluidics offers an efficient alternative to bulk methods, producing nanomaterials with improved size uniformity, morphology control, and overall quality [32,33,34].

Thus, in the context of water pollution, associated public health concerns, and the limitations of conventional water treatment strategies, there is an urgent need to develop new, efficient water decontamination approaches. In line with this need, the present study focuses on developing a new material with potential applications in water decontamination, aiming to enhance adsorption performance and provide a potentially safe material for environmental remediation. Specifically, a silica-based aerogel functionalized with titanium and incorporated with magnetite nanoparticles (Fe_3_O_4_-SA) functionalized with salicylic acid (AG-Ti@Fe_3_O_4_-SA) was obtained using a microfluidic platform, exhibiting a porous structure with integrated magnetite nanoparticles and titanium ions within the aerogel matrix, as confirmed by XRD, FT-IR, RAMAN, SEM, TEM, and BET analyses. The material demonstrated variable decontamination efficiency, ranging from 16 to 100%, indicating a strong affinity for organophosphorus compounds and moderately polar compounds. The biological assays indicate a cell-type-dependent biological response of the material and the potential to develop a selective adsorbent for water decontamination applications.

## 2. Results and Discussion

Given the global crisis of water pollution caused by pesticides and the need to develop innovative, effective materials to address this issue, this study proposes a new composite aerogel, AG-Ti@Fe_3_O_4_-SA, with improved properties, as a new strategy for decontamination applications. To improve its features, the material was synthesized via microfluidics, which helped control reaction parameters (including precipitation and homogeneous mixing), resulting in a uniform porous network of interconnected pores and a uniform dispersion of Fe_3_O_4_ within the aerogel matrix.

The chemical composition of AG-Ti@Fe_3_O_4_-SA is a key factor in its decontamination performance. In this regard, the sodium trisilicate-derived silica network confers high surface area to the aerogel, while the alginic acid sodium salt provides flexibility and abundant oxygen-containing active sites. CTAB contributes to the aerogel porosity, and the introduction of titanium and Fe_3_O_4_ nanoparticles provides supplementary adsorption sites. Moreover, the nanoparticles are embedded as dispersed magnetic domains, which contribute to structural heterogeneity and promote magnetic recovery after decontamination. To provide as much information as possible about its physicochemical properties, the material was characterized using XRD, FT-IR, Raman, SEM, TEM, and BET. In addition, the performance of the aerogel under study for water purification was evaluated using HR-MS FT-ICR, and its biocompatibility was assessed through several biological assays.

### 2.1. Aerogel Characterization

**Figure 1:**
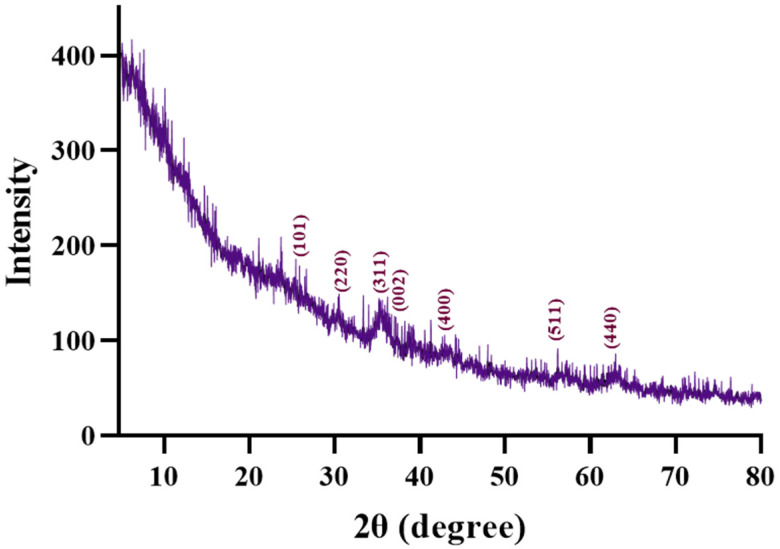
XRD diffractogram of composite aerogel AG-Ti@Fe_3_O_4_-SA.

Figure 1 represents the diffractogram of the AG-Ti@Fe_3_O_4_-SA aerogel, which shows distinct diffraction peaks for both silica and iron oxide. In the ~20–30° interval, a specific large halo specific to amorphous silica can be observed. Also, the XRD analysis confirms the successful integration of Fe_3_O_4_ and titanium in the aerogel matrix. Diffuse reflections associated with the spinel phase of Fe_3_O_4_ (ASTM 01-075-160) identified at 2theta angles of ~30°, ~35°, ~43°, ~54°, ~57°, and ~63° correspond to the (220), (311), (400), (422), (511), (440) planes, indicating the formation of well-dispersed nanoparticles in the aerogel matrix. The intensity associated with titanium at 2theta angles of ~25° and ~37° is weak, partially overlapped by the amorphous background, suggesting that it is well integrated into the aerogel.

**Figure 2:**
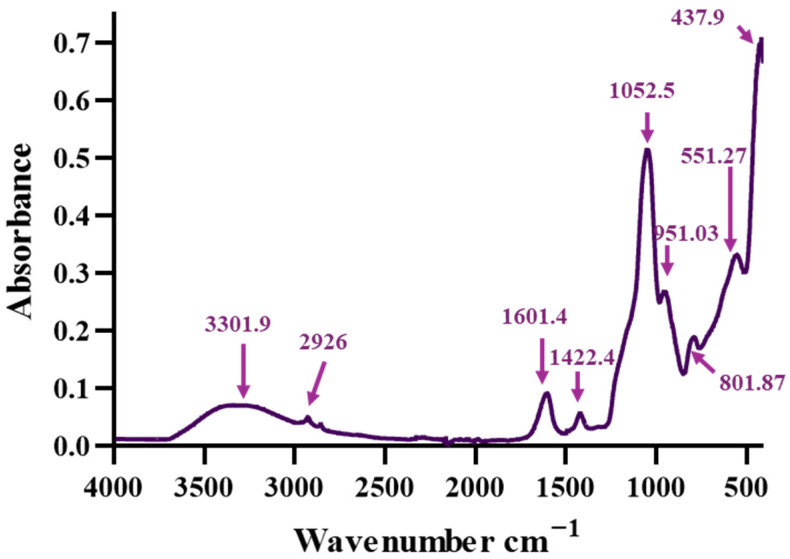
FT-IR spectrum of composite aerogel AG-Ti@Fe_3_O_4_-SA.

The FT-IR spectrum of the composite aerogel AG-Ti@Fe_3_O_4_-SA obtained through the microfluidic platform is presented in Figure 2. The value around 3301 cm^−1^ is attributed to O–H stretching vibrations from silanol (Si–OH) groups, alginate, and to the salicylic acid used in the functionalization of the Fe_3_O_4_ nanoparticles [35,36,37,38]. The peaks at 2926, 1601.4, and 1422.4 cm^−1^, associated with CH_2_ asymmetric stretching, C=O stretching, and COO^−^ groups, respectively, are present due to both alginate (used in the aerogel matrix) and salicylic acid from the surface of incorporated Fe_3_O_4_ nanoparticles [36,37,38]. The bands observed at ~1052, ~801, and ~437 cm^−1^ are characteristic of the Si–O–Si stretching mode, confirming the formation of the silica aerogel matrix. Moreover, the signal at ~951 cm^−1^ is associated with Si–OH or Si–O–Ti, suggesting titanium bonding to the silica network and good dispersion [39]. The peak visible at ~437 cm^−1^ can also be associated with Ti–O [40]. The signal around 551 cm^−1^ is characteristic of Fe–O vibrations in magnetite, confirming the presence of the Fe_3_O_4_ phase in the composite [41].

**Figure 3:**
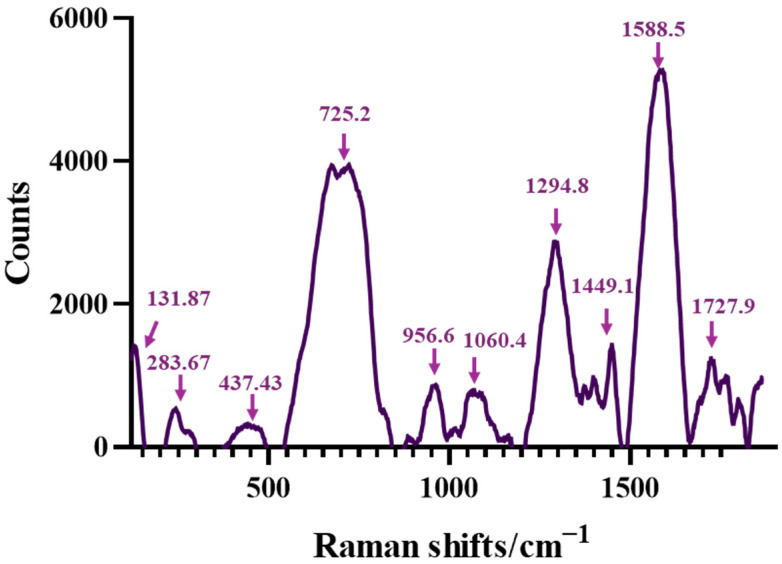
RAMAN spectra for composite aerogel AG-Ti@Fe_3_O_4_-SA.

The RAMAN spectrum of AG-Ti@Fe_3_O_4_-SA is shown in Figure 3, and the analysis was performed to provide additional information on the composite aerogel’s key functional groups. Thus, in the spectrum, distinct bands are observed at ~283.7, 437.4, 725.2, 956.6, 1060.4, 1294.8, 1449.1, 1588.5, and 1727.9 cm^−1^. The intense bands observed at ~1588 cm^−1^ can be attributed to the stretching band of the aromatic ring from salicylic acid or to the alginate’s asymmetric COO^−^, while ~1449.1 cm^−1^ band is associated with the C=C stretching of the benzene ring, and the ~1727.9 cm^−1^ stretching band corresponds to C=O vibrations of the carboxylic acid group of the salicylic acid [42,43]. The band around 1060.4 cm^−1^ is typical of the C–O vibrations of polysaccharides (alginate) [43]. The signal observed at ~437,4 cm^−1^ is characteristic of Si–O–Si deformation modes of the silica lattice [44,45]. The band observed at ~956.6 cm^−1^ is associated with Si–OH vibrations and/or Si–O–Ti bonds, the region around 960–980 cm^−1^ being associated with these bonds in previous works [45,46]. In the RAMAN spectrum presented below, a pronounced band at ~667.8 cm^−1^ is also observed, which is characteristic of the A_1_g mode of magnetite (Fe_3_O_4_), considered to be the fingerprint of the inverse spinel structure of the magnetite and attributed to the symmetric stretching vibration of Fe–O bonds [47,48]. The presence of this band confirms the formation and maintenance of the unaltered magnetite phase within the composite material. Moreover, the literature reported that prominent bands at ~300, ~500, and ~660 cm^−1^ are also characteristic of magnetite [49]. The slight shift in the typical values of the bulk Fe_3_O_4_ material suggests that the Fe_3_O_4_ nanoparticles have been integrated into the aerogel matrix.

**Figure 4:**
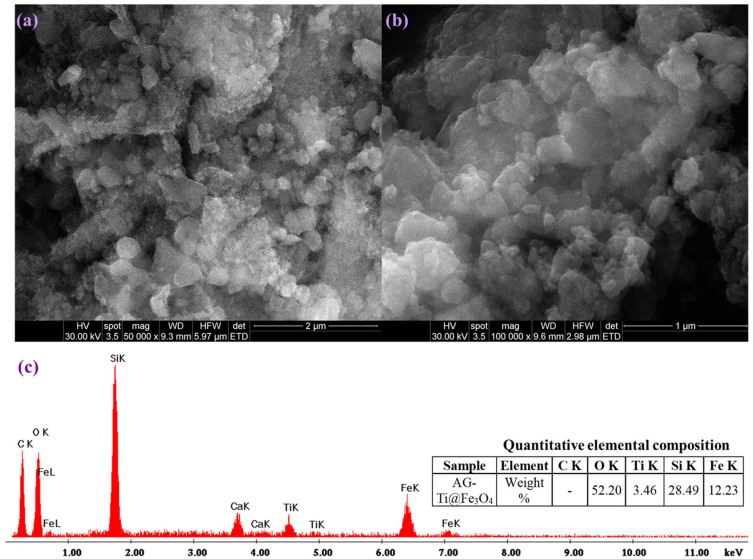
SEM micrographs at (**a**) 50,000×, (**b**) 100,000×, and (**c**) EDS results of composite aerogel AG-Ti@Fe_3_O_4_-SA.

SEM micrographs for the AG-Ti@Fe_3_O_4_-SA composite aerogel are shown in Figure 4. The material presents a sponge-like morphology composed of submicron aggregates of nanometric primary particles. Furthermore, the aerogel exhibits a porous structure with locally denser areas, associated with Fe_3_O_4_ nanoparticle loading, suggesting successful incorporation and partial agglomeration within the aerogel matrix. In addition, the EDS results confirm the material’s elemental composition, revealing the presence of aerogel’s specific elements, including carbon (C), silicon (Si), oxygen (O), iron (Fe), and titanium (Ti).

**Figure 5:**
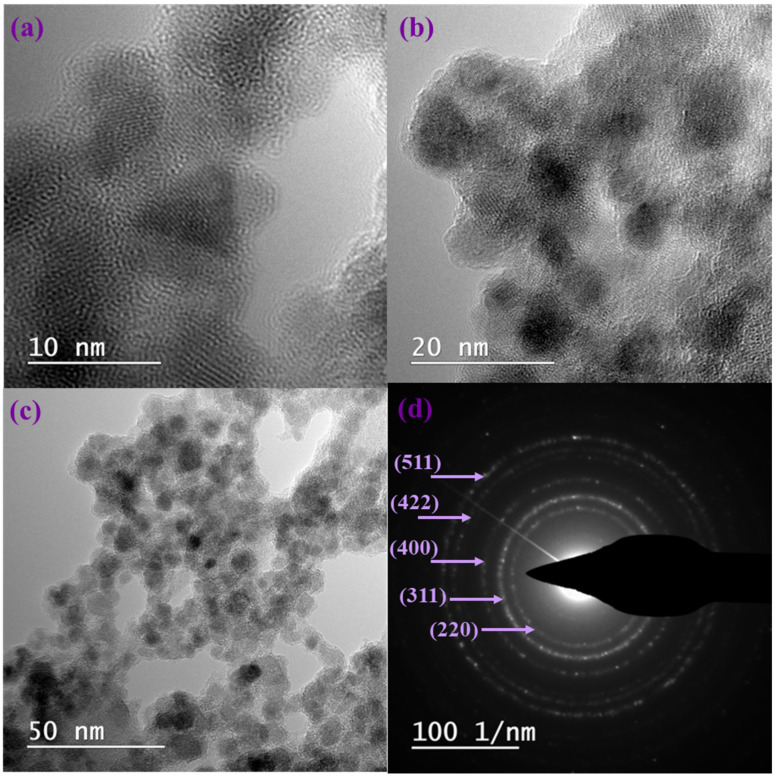
(**a**,**b**) TEM micrographs, (**c**) HR-TEM images, and (**d**) SAED pattern of AG-Ti@Fe_3_O_4_-SA.

Figure 5 presents TEM micrographs of the composite aerogel AG-Ti@Fe_3_O_4_-SA, which reveal the distribution of small quasi-spherical Fe_3_O_4_ nanoparicles. Dark regions correspond to Fe_3_O_4_ nanoparticles, which remain well-embedded and uniformly distributed within the aerogel matrix (indicated by the lighter backgrounds). Moreover, the high-resolution TEM (HR-TEM) image (Figure 5c) shows well-defined lattice fringes, indicating the high crystallinity of the Fe_3_O_4_ nanoparticles. The SAED pattern (Figure 5d) shows distinct concentric rings characteristic of the cubic spinel structure of Fe_3_O_4_, indicating the polycrystalline nature and phase purity of the nanoparticles. Thus, the rings correspond to the (220), (311), (400), (422), and (511) planes of magnetite, confirming the XRD results.

Table 1 summarizes the results of the DLS analysis of the aerogel composite AG-Ti@Fe_3_O_4_-SA. The measurements yield a zeta potential of approximately −12 mV, suggesting limited colloidal stability in aqueous media. On the other hand, their large hydrodynamic radius and increased polydispersity confirm the tendency toward micro-sized agglomeration, characteristic of silica-based aerogels containing Fe_3_O_4,_ which are associated with magnetic interactions between nanoparticles and the intrinsic porous network. Therefore, the DLS results reflect the hydrodynamic behavior of aerogel aggregates in the liquid phase and are not directly comparable to the primary particle sizes observed in SEM and TEM analysis.

In regard to water treatment, the relatively low zeta potential of the AG-Ti@Fe_3_O_4_-SA aerogel is not necessarily detrimental. In this context, for the proposed application, these adsorbent materials used in water treatment processes can be recovered after the adsorption step due to the integration of Fe_3_O_4_-SA nanoparticles. An increased colloidal stability could complicate solid–liquid separation. In AG-Ti@Fe_3_O_4_-SA, the incorporation of Fe_3_O_4_ nanoparticles can facilitate magnetic recovery of the aerogel aggregates, thereby improving separation from treated water. However, evaluating the long-term stability of the AG-Ti@Fe_3_O_4_-SA aerogel in terms of usage cycles, as well as its regeneration efficiency, represents a direction for future research to fully assess its potential for practical reuse.

**Figure 6:**
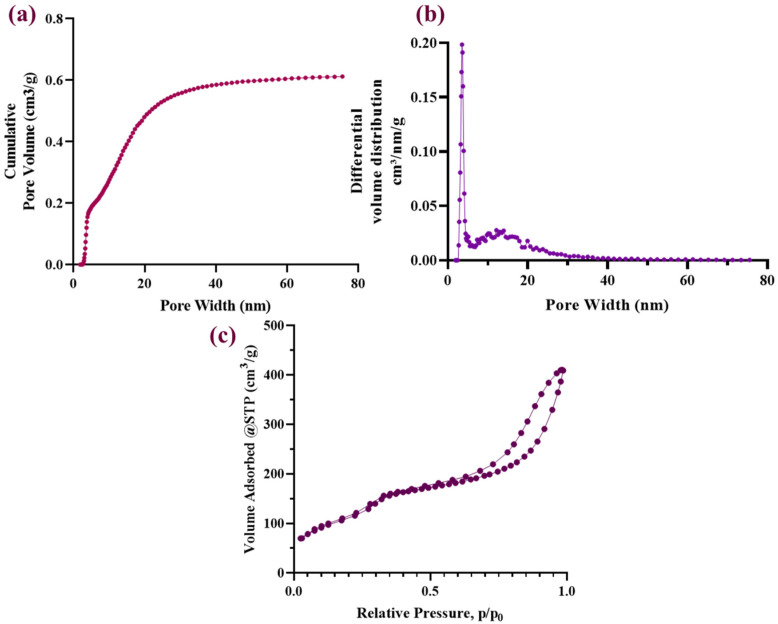
Nitrogen adsorption–desorption characterization of the AG-Ti@Fe_3_O_4_-SA aerogel: (**a**) cumulative pore volume, (**b**) pore size distribution, (**c**) adsorption–desorption isotherm.

For further characterization of AG-Ti@Fe_3_O_4_-SA aerogel in terms of its porosity, Brunauer–Emmett–Teller analysis (BET) (Figure 6) was performed. The results suggest that the AG-Ti@Fe_3_O_4_-SA aerogel pore volume curve (Figure 6a) demonstrated a rapid increase in the cumulative pore volume, ranging between 10 and 15 nm, suggesting that the pore volume is associated with mesopores. Then, the curve gradually approaches a plateau, indicating that most of the pore volume is associated with small mesopores. The total pore volume is ~0.6 cm^3^/g, as shown in Table 2. The pore size distribution (Figure 6b) presents a dominant and sharp peak at 3–4 nm, confirming the fact that AG-Ti@Fe_3_O_4_-SA aerogel is predominantly characterized by small mesopores, but also, to some extent, by larger mesopores (5–20 nm) in lower proportions. These findings confirm the formation of the porous network in the aerogel. Moreover, the aerogel’s porous structure can facilitate the adsorption of small molecules. In contrast, the material can potentially limit the diffusion of bulkier species due to steric constraints within the pore network. The N_2_ adsorption–desorption isotherm (Figure 6c) showed that the AG-Ti@Fe_3_O_4_-SA aerogel presents a typical type IV profile with a well-defined hysteresis loop. This profile is characteristic of mesoporous materials, confirming that AG-Ti@Fe_3_O_4_-SA aerogel presents interconnected mesopores. These features are associated with its specific surface area (~436 m^2^/g) (Table 2) and the pore size distribution centred in the mesopore range.

### 2.2. Decontamination Performance

HR-MS FT-ICR analysis was performed to provide quantitative data on the decontamination performance of AG-Ti@Fe_3_O_4_-SA, as shown in Table 3. The adsorption tests were performed as screening-level experiments under fixed conditions and at low pesticide concentrations (ppb), which are representative of real environmental conditions in contaminated water sources. In this context, the analysis demonstrated that the composite aerogel AG-Ti@Fe_3_O_4_-SA selectively removed the tested pesticides. The performance ranges from 16% to 100%. The best decontamination performance was obtained with chlorpropham (100%), followed by triazophos (98%), fenson (85%), fenthion (77%), and chlorthal-dimethyl (75%). Cypermethrin and fenpropathrin showed lower removal capacities (<20%). These results suggest that the selectivity of the obtained material is governed by a combination of molecular size, hydrophobicity, and specific surface chemistry interactions. However, the calculated adsorption capacities (mg/g) are relatively low and should not be directly compared with values reported in optimized adsorption studies conducted at higher concentrations.

**Figure 7:**
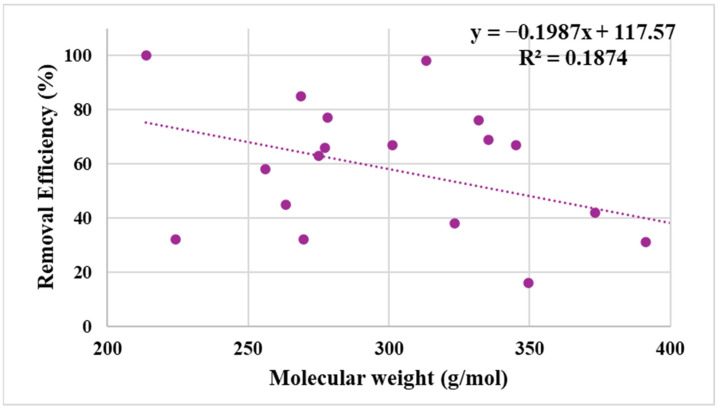
Relationship between molecular weight and removal efficiency of pesticides by AG-Ti@Fe_3_O_4_-SA aerogel.

Moreover, Figure 7 explores the influence of the molecular properties of the pesticides used in this study on the adsorption behavior and the removal efficiency of AG-Ti@Fe_3_O_4_-SA. This analysis confirms the results provided in Table 2. Pesticides with larger and bulkier molecules tend to exhibit lower removal efficiencies. This performance can be explained by the steric limitations and reduced diffusion rates within the porous aerogel network. In contrast, smaller molecules generally showed higher adsorption efficiencies, suggesting easier access to internal adsorption sites within the aerogel structure. However, the correlation is not strictly linear, indicating that adsorption is governed by multiple factors, including molecular size, hydrophobicity, and specific interactions between pesticide functional groups and the aerogel surface.

**Figure 8:**
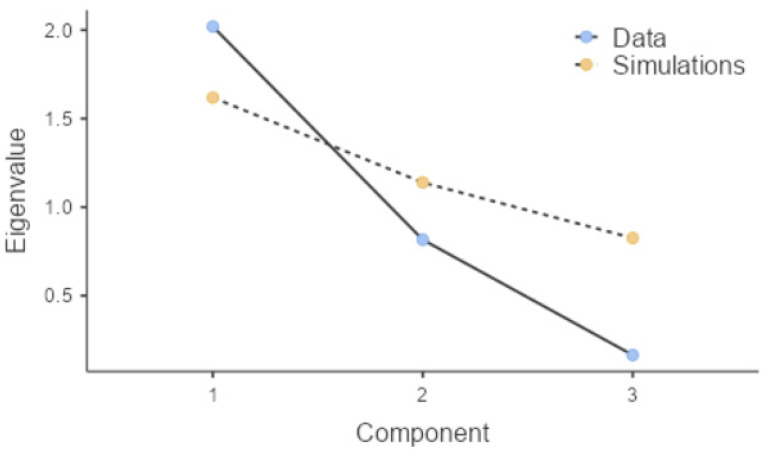
Principal component analysis representation of the relationship between molecular descriptors (LogP and molecular weight) and removal efficiency. Created with Jamovi Software Version 2.7.24.0 [50].

The observation is further confirmed by the principal component analysis (PCA) results (Figure 8). For this analysis, standardized variables such as LogP, molecular weight, and removal efficiency were used. In this context, PCA revealed that the molecular weight and LogP of the pesticides are associated with the primary component (PC1), a statistically significant association. In contrast, the second component (PC2) exhibits additional variability not explained by the same parameters, suggesting specific molecular features and interactions. The third component (PC3) accounts for a small proportion of the variance and is not relevant for further interpretation. PC1 reflects the combined effect of molecular size and hydrophobicity; PC2 likely represents more subtle differences in molecular structure and specific interactions with the adsorbent surface; and PC3 contributes only marginally to the total variance and is not chemically meaningful. Together, these results confirm that the adsorption process is governed by multiple interacting factors rather than a single molecular descriptor.

In the context of water depollution, the adsorptive properties of aerogels are driven by synergistic multi-interaction mechanisms, including chemisorption, hydrogen bonding, electrostatic interactions, π-π stacking, van der Waals forces, and hydrophobic interactions [51,52]. For the proposed aerogel, the silica–alginate framework provides abundant oxygen-containing functionalities (Si–OH, –COO^−^, –OH) that interact with organic molecules via hydrogen bonding and dipole–dipole interactions, as reported in the literature for bulk and silica–alginate composite materials [53,54,55]. Moreover, the incorporation of titanium into the silica network modifies the surface chemistry and leads to the formation of Si–O–Ti bonds, which can improve surface polarity and adsorption affinity, potentially favoring electrostatic interactions and hydrogen bonding, thereby enhancing interactions with electron-donor functional groups [56,57,58]. Fe_3_O_4_ nanoparticles can enhance electron transfer and electrostatic interactions with aromatic dyes and pesticides, and their functionalization with salicylic acid confers good dispersability and long-term stability [52,59]. Additionally, salicylic acid can provide phenolic and carboxyl groups that serve as active donor sites capable of forming hydrogen bonds and coordination interactions with target species [60].

Nevertheless, while the use of TiO_2_ has not been studied in relation to the adsorption of pollutants on its surface, but rather for its photocatalytic properties, adsorption on its surface represents an important step in enabling its photocatalytic properties [61].

In this context, the adsorption of pesticide molecules onto the TiO_2_ surface is a prerequisite for subsequent photocatalytic reactions. In any case, exploring the photocatalytic properties of the AG-Ti@Fe_3_O_4_-SA aerogel is a future research direction that may improve its efficiency in applications such as water decontamination from pesticides. In this regard, our FT-IR and RAMAN analyses provide insights regarding the possible presence of titanium oxides. Thus, doping the aerogel with titanium can promote an adsorption mechanism that was reported in the literature. Studies indicate that TiO_2_ possesses a large number of active sites associated with surface defects and water molecules that can occupy vacant oxygen sites that form –OH groups adsorbed on the surface, thereby improving hydrophilicity and facilitating adsorption, thereby enhancing the interaction between the TiO_2_ surface and the adsorbed pesticide molecules, being a key factor for the efficiency of photocatalytic degradation [62,63].

Moreover, despite the fact that DLS analysis indicates a tendency for relatively large aggregates to form in aqueous suspension, this phenomenon cannot necessarily be associated with a significant reduction in the accessible adsorption surface area of the aerogel. In porous aerogel systems, adsorption occurs primarily within the internal pore network rather than on the external surface. Thus, although aggregates form, the interconnected porosity of aerogels remains accessible to small pesticide molecules. However, these findings cannot entirely rule out the possibility that aggregation may impose diffusion limitations, particularly for bulky pesticides with high hydrophobicity. This detail may partially explain the lower decontamination efficiencies observed for compounds such as cypermethrin and fenpropathrin. Furthermore, the relatively high removal efficiencies observed for smaller, polar pesticides in decontamination performance tests at a contact time of 30 min may suggest that mass-transfer limitations are not the determining factor under these test conditions. Instead, for bulky pesticides with increased hydrophobicity, diffusion limitations within the porous network may reduce decontamination capacity.

AG-Ti@Fe_3_O_4_-SA exhibits competitive and selective decontamination efficiency ranging from 16 to 100%, and also has evident limitations for other pesticides, as shown in Table 4. Comparing these results with our previous studies suggests that the decontamination performance of the aerogels depends on the molecular structure of the pesticides.

Furthermore, as previously mentioned, the studied AG-Ti@Fe_3_O_4_-SA aerogel exhibits a selective profile, showing a stronger affinity for certain organophosphorus pesticides, rather than the uniformly high removal efficiency observed in our previously obtained materials. This may be attributed to the presence of titanium in the aerogel’s composition. Thus, some studies have reported that, in adsorption experiments, the hydroxyl groups on the TiO_2_ surface interacted with the P=O groups in organophosphorus pesticides. According to the research, this interaction led to molecular adsorption, without the immediate chemical decomposition of the organophosphorus compound [67]. Moreover, the capacity of TiO_2_ to adsorb organophosphorus pesticides can be attributed to Lewis acid sites, which promote the donation of electron density from the oxygen atoms of the phosphate group to surface titanium atoms. This interaction weakens the P–O ester bond within the molecule and may facilitate its cleavage during hydrolysis [68].

For organic pesticides, such as paraoxon-ethyl, propyzamide, EPN, and pyrazophos, the efficiency of AG-Ti@Fe_3_O_4_-SA in their decontamination falls within the ranges reported in our previous study about magnesium magnetic silica-based aerogel. This result indicates comparable performance and confirms the dominant roles of coordination interactions and adsorption within the mesoporous network. A significant result for triazophos is observed with AG-Ti@Fe_3_O_4_-SA, exceeding the reported range in some previous studies, which can be attributed to the superior affinity arising from the contribution of Ti sites and the synergy with Fe_3_O_4_ nanoparticles. In contrast, for strongly hydrophobic and bulky pesticides such as cypermethrin and fenpropathrin, it is suggested that, in the AG-Ti@Fe_3_O_4_-SA system, they fall at the lower end of previously reported ranges, which can be attributed to diffusion limitations and a reduced contribution from hydrophobic interactions. As shown in Table 3, both systems exhibit strong affinities for additional pesticides, suggesting multimodal adsorption behavior. AG-Ti@Fe_3_O_4_-SA shows more pronounced selectivity, with notable efficacy for organophosphorus pesticides, compared to the aerogel from the previous study, which shows a more generalist adsorptive behavior.

Compared with the previously reported magnetic silica aerogel [65], the herein reported AG-Ti@Fe_3_O_4_-SA aerogel shows high efficiency for certain compounds, achieving complete or near-complete elimination of chlorpropham and triazophos, and reduced performance against other hydrophobic pesticides. This indicates that its overall performance is less robust than that of our previously reported magnetic aerogel. The magnetic silica-based aerogel exhibits uniform adsorptive properties, with efficiency ranging from 34.8% to 93.7% for the tested pesticide. These data suggest that the material obtained exhibits a much broader affinity and more balanced adsorption mechanisms than those of AG-Ti@Fe_3_O_4_-SA aerogel.

Also, comparing AG-Ti@Fe_3_O_4_-SA with the Mg/Fe-LDH–silica–Fe_3_O_4_ material [66], it can also be observed that AG-Ti@Fe_3_O_4_-SA has a selective character, but much more variable than Mg/Fe-LDH–silica–Fe_3_O_4_. Thus, Mg/Fe-LDH–silica–Fe_3_O_4_ exhibited high and relatively uniform efficiencies, ranging from approximately 73.7% to 100% for the tested pesticide mixture. Compared to AG-Ti@Fe_3_O_4_-SA aerogel, Mg/Fe-LDH–silica–Fe_3_O_4_ pesticide adsorption efficiency exceeded the 80% threshold, and complete elimination (100%) was achieved for Chlorpropham. Thus, for AG-Ti@Fe_3_O_4_-SA, the efficiencies are more dispersed and depend on the type of pesticide. Excellent performance is observed for some molecules, such as Chlorpropham (100%) and Triazophos (98%), comparable to those of Mg/Fe-LDH–silica–Fe_3_O_4_. At the same time, the affinity of AG-Ti@Fe_3_O_4_-SA aerogel for organophosphorus compounds such as fenitrothion (66%) and fenthion (77%) is more pronounced, with moderate efficiency. It is also confirmed that the AG-Ti@Fe_3_O_4_-SA aerogel exhibits limited adsorption of more hydrophobic and bulky pesticides (e.g., cypermethrin 18%, fenpropathrin 16%, permethrin 31%), underscoring the need for further optimization to effectively remove these pesticides from aqueous samples.

Overall, these findings suggest that the dominant mechanism for AG-Ti@Fe_3_O_4_-SA depends on pore accessibility, as well as on π-π interactions and hydrogen bonds. Thus, the material favors polar and organophosphorus pesticides, which contain heteroatoms (O, N, S) that can form hydrogen bonds, dipole–dipole interactions, or weak coordination. In contrast, bulky, strongly hydrophobic pesticides are adsorbed more weakly, probably due to diffusion limitations within the pores and unfavorable surface interactions. The incorporation of titanium creates sites that facilitate interactions with electron-donating groups in pesticides, such as P=O, C=O, or heteroatoms, thereby explaining the affinity for polar and organophosphorus compounds. Furthermore, the functionalization of Fe_3_O_4_ nanoparticles with salicylic acid can enhance the material’s selectivity through π-π interactions and additional hydrogen-bonding interactions.

### 2.3. Biological Assays

For the in vitro investigation, a concentration of 5 μg/mL of the composite aerogel was selected, as it could represent an environmentally relevant exposure level after applications in water purification strategies, as small residual amounts may remain in treated water and potentially interact with human tissues. Such exposure could occur through direct skin contact during handling or, less frequently, through accidental ingestion or occupational scenarios. Therefore, the use of human keratinocytes is helpful for assessing cutaneous exposure, and kidney-derived cells simulate systemic effects, given the kidney’s essential role in filtering and eliminating circulating xenobiotics. In this way, the chosen concentration of 5 μg/mL could provide a balanced experimental condition, appropriate for evaluating both environmental relevance and potential toxicological implications.

**Figure 9:**
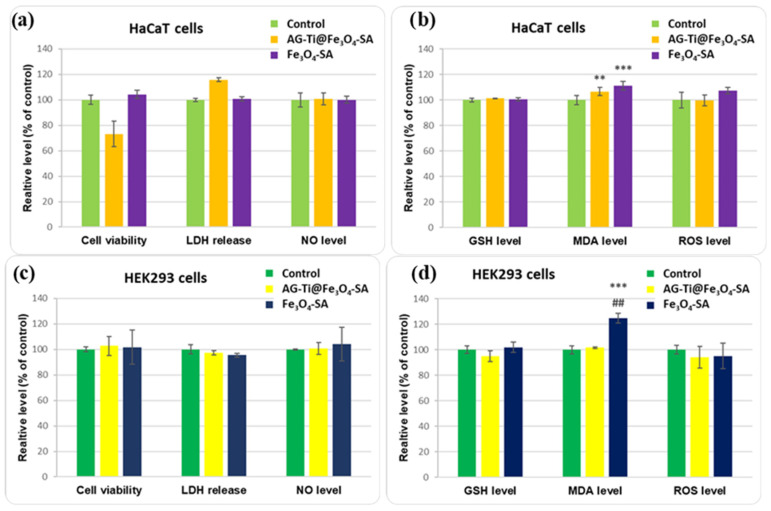
Comprehensive evaluation of the biological effects induced by the AG-Ti@Fe_3_O_4_-SA composite aerogel and Fe_3_O_4_-SA nanoparticles in HaCaT (**a**,**b**) and HEK293 (**c**,**d**) cells after 48 h of exposure. Biocompatibility was assessed by measuring cell viability (MTT assay), LDH release, and nitric oxide (NO) production, while oxidative stress was evaluated by quantifying reduced glutathione (GSH), malondialdehyde (MDA), and intracellular reactive oxygen species (ROS). The results are expressed as mean ± standard deviation of three independent experiments and presented relative to controls. Statistical significance is indicated as ** *p* < 0.01 and *** *p* < 0.001 vs. control, and ## *p* < 0.01 vs. Fe_3_O_4_-SA nanoparticles.

A comprehensive assessment of biocompatibility and oxidative stress in HaCaT (Figure 9a,b) and HEK293 (Figure 9c,d) cells after 48 h exposure to AG–Ti@Fe_3_O_4_–SA composite aerogel and Fe_3_O_4_–SA nanoparticles is depicted in Figure 6. Regarding the biocompatibility of HaCaT cells (Figure 9a), viability was reduced by 26% after incubation with AG–Ti@Fe_3_O_4_–SA compared to the control, whereas lactate dehydrogenase (LDH) release was 15% higher than the control, suggesting mild membrane damage. These results indicate a moderate cytotoxic response in keratinocytes under the tested conditions. Importantly, NO production did not appear substantially altered, indicating the absence of a pronounced inflammatory response. However, the Fe_3_O_4_–SA nanoparticles did not affect the keratinocytes’ viability. Furthermore, oxidative stress markers (Figure 9b) show a significant increase in MDA levels following exposure to the composite aerogel or Fe_3_O_4_–SA nanoparticles compared to control cells, indicating a certain degree of lipid peroxidation induced during the 48 h. ROS levels were slightly elevated only for Fe_3_O_4_–SA nanoparticles, while GSH levels remained relatively close to control values for both types of samples, suggesting that the antioxidant defense system was largely preserved.

In HEK293 cells (Figure 9c), cell viability was close to control values for both samples, with no significant change in LDH release. NO levels were slightly increased for Fe_3_O_4_–SA, but without a clear indication of inflammatory activation. The measurement of oxidative stress parameters (Figure 9d) revealed no changes compared to control for composite aerogel, but showed a more pronounced increase in MDA levels after incubation with Fe_3_O_4_–SA, suggesting enhanced lipid peroxidation in this cell line. Regarding GSH levels and ROS production, only slight variations were observed, which did not exceed control values. These findings indicate that oxidative imbalance is primarily reflected in lipid peroxidation rather than in global ROS accumulation.

**Figure 10:**
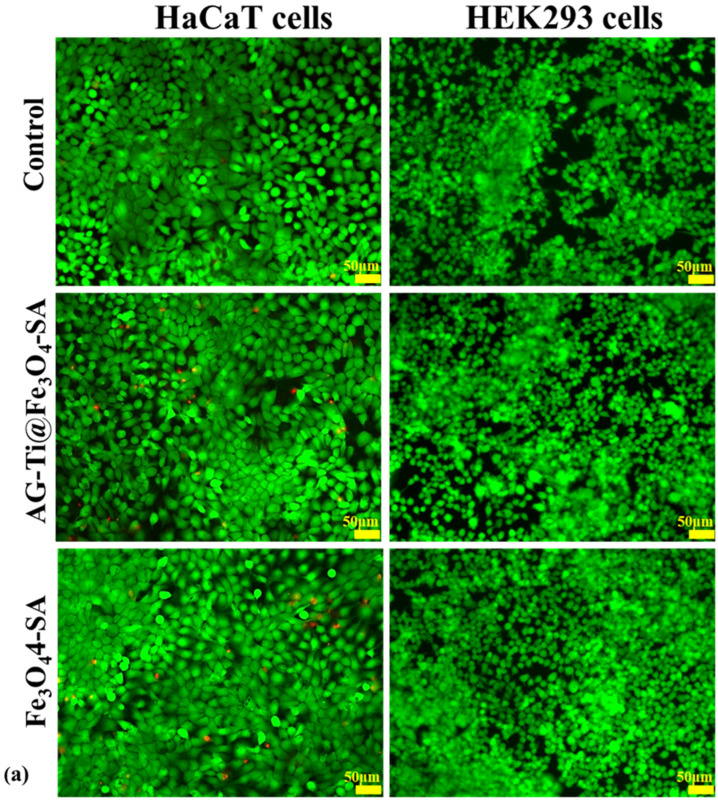

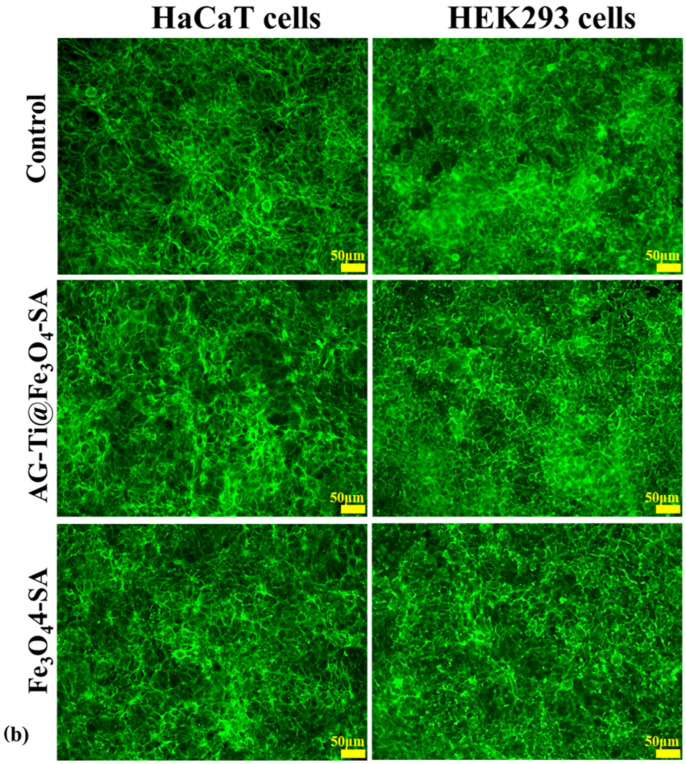
Representative fluorescence images of HaCaT and HEK293 cells after 48 h incubation with the AG-Ti@Fe_3_O_4_-SA composite aerogel and Fe_3_O_4_-SA nanoparticles: (**a**) Live/dead staining was performed using calcein-AM (green fluorescence showing viable cells) and ethidium homodimer-1 (red fluorescence showing non-viable cells). (**b**) The actin cytoskeleton was visualized by FITC–phalloidin staining (green fluorescence), which highlighted F-actin. Scale bar: 50 µm for all images.

The fluorescence microscopy images in Figure 10a illustrate the viability and morphology of HaCaT and HEK293 cells after 48 h exposure to AG–Ti@Fe_3_O_4_–SA and Fe_3_O_4_–SA samples, compared to control cells. In all experimental conditions, a high proportion of metabolically active cells and only sporadic non-viable cells were visible, suggesting limited membrane damage, except for the exposure of human keratinocytes to AG–Ti@Fe_3_O_4_–SA, where more dead cells were noticed, labeled in red with propidium iodide, supporting the quantitative data (MTT and LDH assays).

Importantly, no major differences in cell morphology were observed between exposed and control wells in either cell line. HaCaT cells maintained their typical cobblestone-like epithelial morphology, while HEK293 cells preserved their characteristic polygonal/rounded appearance and confluence.

Figure 10b presents the organization of the actin cytoskeleton in HaCaT and HEK293 cells after 48 h of treatment. In both cell lines, F-actin filaments appeared well distributed and organized, forming a continuous cytoskeletal network comparable to that of control cells. No evident signs of cytoskeletal disruption, actin condensation, fragmentation, or loss of structural integrity were observed in the treated groups. Cell shape and spreading remained preserved, and there was no indication of cytoskeletal collapse or stress fiber disorganization, which are commonly associated with cytotoxic or pro-apoptotic effects.

Taken together, these findings further support a good in vitro biocompatibility profile of the composite aerogel under the tested conditions.

Overall, the biological assays (Figure 9 and Figure 10) indicate a cell-type-dependent biological response, characterized by a moderate reduction in viability in HaCaT keratinocytes but good tolerance in HEK293 cells. These biological responses can be attributed to the aerogel’s chemical composition. The mild viability reduction and slightly increased LDH observed in HaCaT cells may be associated with the presence of titanium in the aerogel composition, while Fe_3_O_4_–SA nanoparticles showed minimal impact on cell survival. In contrast, both AG-Ti@Fe_3_O_4_-SA and Fe_3_O_4_–SA nanoparticles maintained viability close to control levels in HEK293, demonstrating good tolerance in this cell line. Moreover, stable ROS and GSH levels suggest that the AG-Ti@Fe_3_O_4_-SA aerogel matrix limits titanium’s intrinsic oxidative potential. These findings are consistent with reports in the literature supporting the low-to-moderate intrinsic toxicity of silica-based aerogels [52,69] and the low toxicity of titanium and its oxides (TiO_2_) [70]. In addition, according to ISO 10993-5 [71] guidelines for in vitro cytotoxicity testing, the HaCaT cell viability remained above 70% of control levels, which is commonly considered the threshold for non-cytotoxic responses. Considering the preserved cell morphology, cytoskeletal organization, and antioxidant defense parameters, the observed reduction in keratinocyte viability suggests a moderate, localized cellular response rather than extensive cytotoxicity.

Despite our demonstration of the biocompatibility of the AG-Ti@Fe_3_O_4_-SA aerogel, which supports its applicability in water depollution and environmental remediation, the toxicity and environmental impact of aerogel-based materials remain major limiting factors for their real-world applications. In this context, further in-depth studies are necessary to ensure their safe use and eventual large-scale deployment.

## 3. Conclusions

In this work, a novel titanium-functionalized magnetic silica aerogel (AG-Ti@Fe_3_O_4_-SA) was successfully synthesized via microfluidics. The physico-chemical characterization strategies confirmed the formation of a porous structure, with uniform incorporation of Fe_3_O_4_ nanoparticles and effective titanium functionalization within the silica–alginate matrix. The removal of pesticides was demonstrated using HR-MS FT-ICR, indicating the material’s competitive and selective pesticide-removal performance, with high affinity for chlorpropham and triazophos and limited adsorption of hydrophobic pesticides. Biological assays demonstrated an overall favorable in vitro biocompatibility of the material, at environmentally relevant exposure levels, with moderate effects observed in HaCaT cells and good tolerance in HEK293 cells. These findings support the potential applicability of the developed aerogel for water decontamination.

## 4. Materials and Methods

### 4.1. Aerogel Synthesis

The AG-Ti@Fe_3_O_4_-SA aerogel composite was synthesized using the following precursors: sodium trisilicate, alginic acid sodium salt from brown algae, cetyltrimethylammonium bromide (CTAB), ammonium chloride, ethanol, and titanium butoxide (Sigma Aldrich Merck, Darmstadt, Germany), sodium hydroxide (Lach-Ner, Tavarni, Czech Republic).

**Figure 11:**
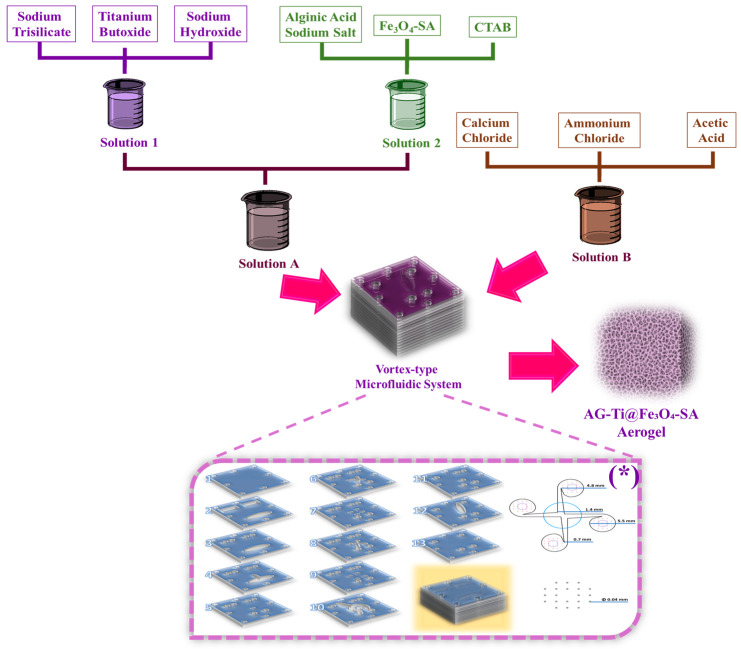
Schematic representation of AG-Ti@Fe3O4-SA aerogel synthesis through a vortex-type microfluidic system. The image noted with (*) is reprinted from an open-access source [73].

Aerogel synthesis involves four solutions (Figure 11), as follows: Solution 1 was obtained by dissolving sodium trisilicate (60 g), NaOH (3.5 g), and titanium butoxide (0.5 mL) in 1000 mL ultrapure water; Solution 2 was prepared by dissolving alginic acid sodium salt (0.1 g) and CTAB (0.5 g) in 300 mL ultrapure water, followed by the addition of a suspension of Fe_3_O_4_-SA nanoparticles (50 mL); Solution A was formed by mixing 150 mL of Solution 1 with 150 mL of Solution 2; Solution B was prepared by dissolving CaCl_2_ (20 g) and NH_4_Cl (2 g) in 400 mL of ultrapure water, then adding acetic acid (10 mL). The aerogel and the incorporated Fe_3_O_4_-SA nanoparticles were obtained using a microfluidic platform, in which an osmotic pump (PSP 220 Pump, Model No. CAR6003, Water Quality Association, Lisle, IL, USA) with a 90 mL/s flow rate was used to inject the two solutions into the vortex-type microfluidic device, as described in our previous studies [65,72]. The obtained aerogel was aged for 24 h, centrifuged at 4500 rpm for 5 min, washed three times with ultrapure water and three times with ethanol, and finally freeze-dried. Throughout all the experimental steps, ultrapure water was used, and all precursors were used as received.

### 4.2. Aerogel Characterization

#### 4.2.1. X-Ray Diffraction (XRD)

Phase composition and crystallinity were determined by using the PANalytical Empyrean diffractometer (PANalytical, Almelo, The Netherlands), equipped with Cu Kα radiation (λ = 1.5406 Å) operated at 45 kV and 40 mA. The diffraction patterns were recorded over 2θ = 5–80° at ω = 0.5°.

#### 4.2.2. Scanning Electron Microscopy (SEM) and Energy Dispersive Spectroscopy (EDS)

The aerogel microstructural features were examined using an Inspect F50 SEM (Thermo Fisher–FEI, Eindhoven, The Netherlands). Thus, the powder was mounted on a carbon tape and introduced into the analysis chamber. Images were acquired with secondary electrons at an accelerating voltage of 30 keV and a spot size of 3.5. Elemental composition was determined using the EDS module coupled to the SEM.

#### 4.2.3. Fourier-Transform Infrared Spectroscopy (FT-IR)

Functional groups present in the AG-Ti@Fe3O4-SA powder sample were identified using a Thermo iN10-MX FTIR spectrometer (Waltham, MA, USA) equipped with a ZnSe crystal. Spectra’s scan was recorded in the 4000–400 cm^−1^ range with 4 cm^−1^ resolution.

#### 4.2.4. RAMAN Spectroscopy

RAMAN spectroscopy was performed on a Renishaw inVia Raman microscope (Wotton-under-Edge, UK) using a 532 nm laser at 50% laser intensity to identify the key functional groups of the AG-Ti@Fe_3_O_4_-SA sample. The measurements were collected over 200–1900 cm^−1^, with spatial resolutions of 0.25–4 μm and 40 accumulations per sample.

#### 4.2.5. Dynamic Light Scattering (DLS)

The zeta potential and hydrodynamic diameter were measured by dynamic light scattering using a DelsaMax Pro-type device (Beckman Coulter, Brea, CA, USA; 532 nm laser). Prior to analysis, the composite powder was dispersed in ultrapure water and sonicated for 10 min at room temperature.

#### 4.2.6. Brunauer–Emmett–Teller Analysis (BET)

The specific surface area of the AG-Ti@Fe_3_O_4_-SA aerogel was determined by BET analysis. For this purpose, a NOVA 800 gas sorption analyzer (Anton Paar QuantaTec, Inc., Boyton Beach, FL, USA) was used. Before measurements were taken, the aerogel sample was degassed at 180 °C for 4 h. Nitrogen adsorption–desorption isotherms were recorded at 77 K over a relative pressure range of *p*/*p*_0_ = 0.005–1.0. To obtain data related to the specific surface area, it was calculated using the BET equation, the total pore volume from the volume of gas adsorbed at *p*/*p*_0_ ≈ 1, and the pore size distribution and mesopore volume were estimated using the BJH model.

#### 4.2.7. High-Resolution Mass Spectrometry (HR-MS)

HR-MS analysis was performed using a Fourier transform ion cyclotron resonance (FT-ICR) spectrometer (SolariX-XR, Bruker Daltonics, Bremen, Germany) equipped with a 15 T magnet. To test the performance of AG-Ti@Fe_3_O_4_-SA, fortified water samples containing a known pesticide mixture were treated with a known mass of aerogel for 30 min. The residual solution was analyzed by direct infusion ESI at a flow rate of 310 µL h^−1^. Operating conditions included N_2_ nebulizer gas at 1.5 L min^−1^, dry gas at 2 L min^−1^ and 210 °C, and a source voltage of 4300 V. Spectra were recorded in the 92–1500 amu range. Adsorption efficiency was assessed by comparing relative signal intensity changes before and after treatment, using fortified standards rather than external calibration curves. Adsorption tests were designed as screening-level evaluations under fixed conditions at low pesticide concentrations.

#### 4.2.8. Biological Assays

The experimental procedures applied in this study are described in detail in Tudorache et al. [66]. Briefly, the potential cytotoxicity of the titanium-functionalized magnetic silica aerogels was evaluated in vitro using two human cell lines: HEK293 (human embryonic kidney cells) and HaCaT (immortalized human keratinocytes). Cells were cultured in DMEM supplemented with 10% fetal bovine serum under standard conditions (37 °C, 5% CO_2_). After trypsinization, they were seeded at a density of 1 × 10^4^ cells/cm^2^ in 25 cm^2^ flasks or 96-well plates and allowed to adhere overnight.

The HEK293 human embryonic kidney cell line was obtained from the American Type Culture Collection (ATCC, cat. no. CRL-1573). The HaCaT human keratinocyte cell line was purchased from Cell Lines Service (CLS, Germany, cat. no. 300493).

Cells were exposed for 48 h to 5 µg/mL of the composite aerogel suspensions prepared in culture medium and sterilized by UV irradiation. Morphological changes were examined under inverted microscopy, and multiple assays were performed to assess biocompatibility and to compare with untreated cells, which served as controls.

Cell viability was determined by the MTT assay (absorbance at 595 nm), nitric oxide production by the Griess reaction (550 nm), and membrane integrity by LDH release (490 nm). Intracellular reactive oxygen species (ROS) were quantified using DCFDA fluorescence (485/515 nm).

Live/dead staining (calcein-AM/ethidium homodimer-1) was used for fluorescence-based viability assessment, while cytoskeletal organization was evaluated by FITC–phalloidin staining of F-actin.

For biochemical analyses, cells were collected after exposure, lysed by sonication, and protein concentration was determined by the Bradford method. Lipid peroxidation was assessed by measuring MDA levels, and GSH content was quantified using the 5,5′-dithiobis(2-nitrobenzoic acid) (DTNB) method.

All experiments were performed in triplicate, and the results were expressed relative to controls. Statistical significance was evaluated using Student’s *t*-test, considering *p* < 0.05 as significant.

## Figures and Tables

**Table 1:** DLS results of aerogel composite AG-Ti@Fe_3_O_4_-SA.

Zeta Potential (mV)	St. Dev.	Hydrodynamic Radius (nm)	St Dev.	Polydispersity (nm)	St. Dev.
−12.36	0.89	1965.93	139.75	1122.13	79.81

**Table 2:** Pore characteristics of AG-Ti@Fe_3_O_4_-SA aerogel.

Surface (m^2^/g)	Pore Volume cm^3^/g	Pore Diameter (nm)
435.87	0.5654	5.1886

**Table 3:** Decontamination performance of AG-Ti@Fe_3_O_4_-SA for various pesticides.

Pesticide	Molecular Formula	Initial Concentration (ppb)	Final Concentration (ppb)	Efficiency (%)	Adsorption Capacity (mg/g)
Alachlor	C_14_H_20_ClNO_2_	0.9894	0.677	32	0.00031
Chlorthal-dimethyl	C_10_H_6_Cl_4_O_4_	1.011	0.241	76	0.00077
Chlorpropham	C_10_H_12_ClNO_2_	0.9925	0.000	100	0.00099
Cypermethrin	C_22_H_19_C_l2_NO_3_	5.0051	4.123	18	0.00088
EPN	C_14_H_14_NO_4_PS	1.0206	0.628	38	0.00039
Fenitrothion	C_9_H_12_NO_5_PS	1.0095	0.342	66	0.00067
Fenpropathrin	C_22_H_23_NO_3_	1.0248	0.864	16	0.00016
Fenson	C_12_H_9_ClO_3_S	0.9976	0.146	85	0.00085
Fenthion	C_10_H_15_O_3_PS_2_	1.2664	0.291	77	0.00098
Mevinphos	C_7_H_13_O_6_P	2.0058	1.372	32	0.00063
Paraoxon-ethyl	C_10_H_14_NO_6_P	0.9804	0.361	63	0.00062
Parathion-methyl	C_8_H_10_NO_5_P	2.0011	1.091	45	0.00091
Permethrin	C_21_H_20_Cl_2_O_3_	4.9982	3.465	31	0.00153
Propyzamide	C_12_H_11_Cl_2_NO	0.9931	0.419	58	0.00057
Prothiofos	C_11_H_15_Cl_2_O_2_PS_2_	1.0059	0.333	67	0.00067
Pyrazophos	C_14_H_20_N_3_O_5_PS	1.0052	0.586	42	0.00042
Tolclofos-methyl	C_9_H_11_Cl_2_O_3_PS	0.9907	0.329	67	0.00066
Triazophos	C_12_H_16_N_3_O_3_PS	0.9948	0.022	98	0.00097
Trifluralin	C_13_H16F_3_N_3_O_4_	0.9893	0.633	69	0.00069

**Table 4:** Reported efficiency ranges for pesticides.

	Efficiency (%)	Magnesium Magnetic Silica-Based Aerogel Efficiency Ranges from Study [64]	Magnetic Silica Aerogel Efficiency [65]	Mg/Fe-LDH–Silica Hybrid Composite Efficiency [66]	This Study
Pesticides					
Alachlor		32–98	-	-	32
Bromopropylate		0–80	-	-	-
Chlorthal-dimethyl		-	63.39	91	76
Chlorpropham		-	48.93	100	100
Cypermethrin		16–67	-	-	18
EPN		0–79	-	-	38
Fenitrothion		-	37.68	79.4	66
Fenpropathrin		25–84	-	-	16
Fensulfothion		28–70	-	-	-
Fenson		-	78.94	94.04	85
Fenthion		-	60.12	87.4	77
Mevinphos		-	57.12	75.32	32
Paraoxon-ethyl		15–81	-	-	63
Parathion-methyl		-	-	-	45
Permethrin		-	-	-	31
Phosalone		46–80	-	-	-
Phosmet		3–93	-	-	-
Propyzamide		34–81	34.84	82.68	58
Pyrazophos		0–82	-	-	42
Prothiofos		-	48.48	89.36	67
Tolclofos-methyl		-	57.21	86.68	67
Tebufenpyrad		31–74	-	-	-
Triazophos		7–79	93.67	98.60	98
Trifluralin		-	45.62	73.72	69

## Data Availability

The original contributions presented in this study are included in the article. Further inquiries can be directed to the corresponding author.
